# Supplementary material for: Mutational landscape of normal epithelial cells in Lynch Syndrome patients
Source: Nat Commun. 2022 May 17;13:2710. doi: 10.1038/s41467-022-29920-2 (PMC9114395; doi:10.1038/s41467-022-29920-2)
Supplement: Supplementary file 1 — Supplementary figures [file 41467_2022_29920_MOESM1_ESM.pdf]

Mutational landscape of normal epithelial cells in Lynch Syndrome patients

Supplementary information

Supplementary Figure 1. Flowchart illustrating the study design.

Supplementary Figure 2. Microdissected crypts in LS patients were clonal.

Supplementary Figure 3. Mutation burden of clonal organoids established from LS patients.

Supplementary Figure 4. ID mutational signatures in the tumour crypts of LS patients.

Supplementary Figure 5. SBS mutational signature extraction and deconvolution.

Supplementary Figure 6. ID mutational signature extraction and deconvolution.

Supplementary Figure 7. Mutational signature validation.

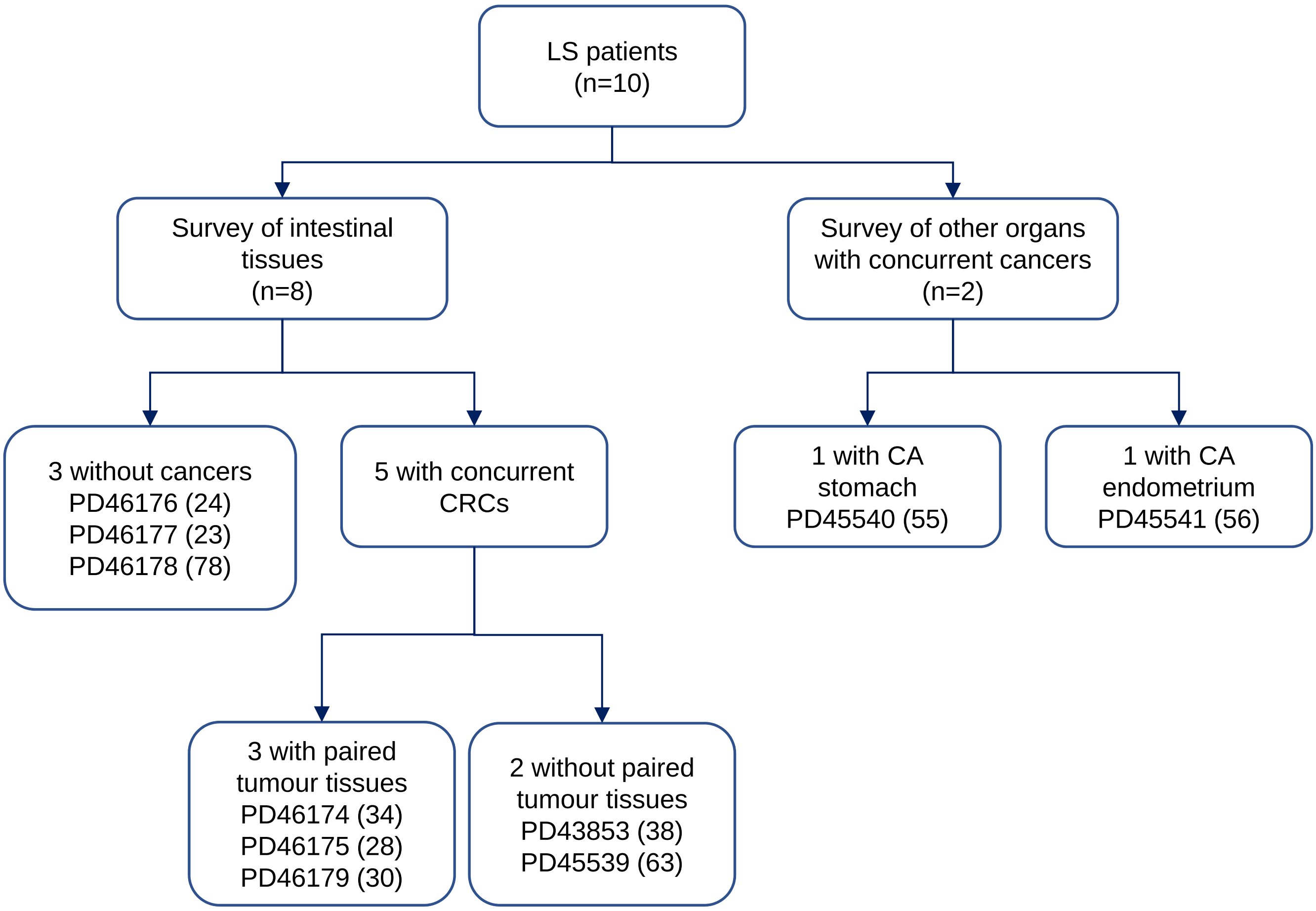

Supplementary Figure 1. **Flowchart illustrating the study design.** A total of 10 LS patients were recruited to the current study. The age of each patient (PD number) is indicated in parentheses.

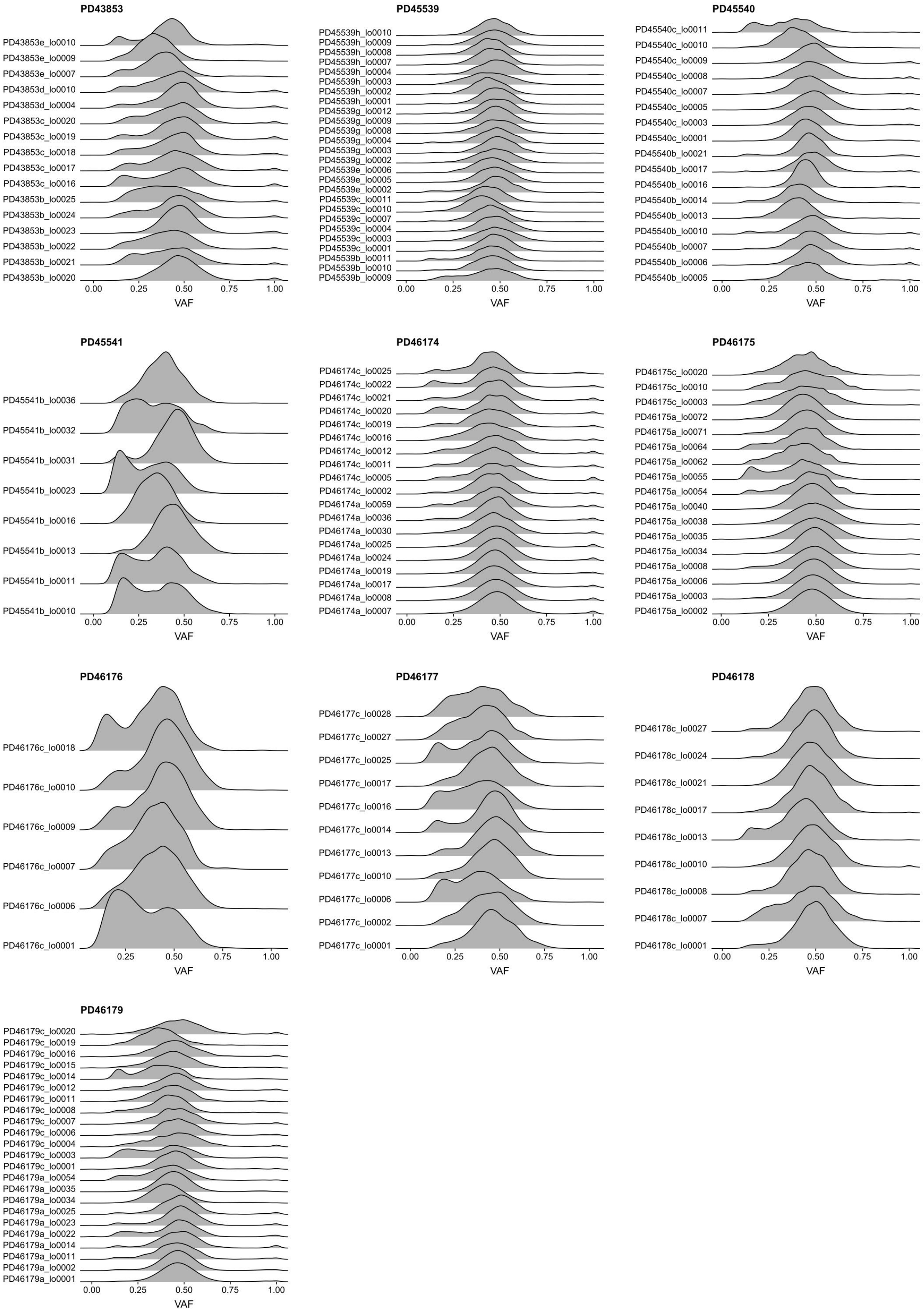

Supplementary Figure 2. Microdissected crypts in LS patients were clonal. Plots showing the variant allele frequency (VAF) distribution of crypts in each patient.

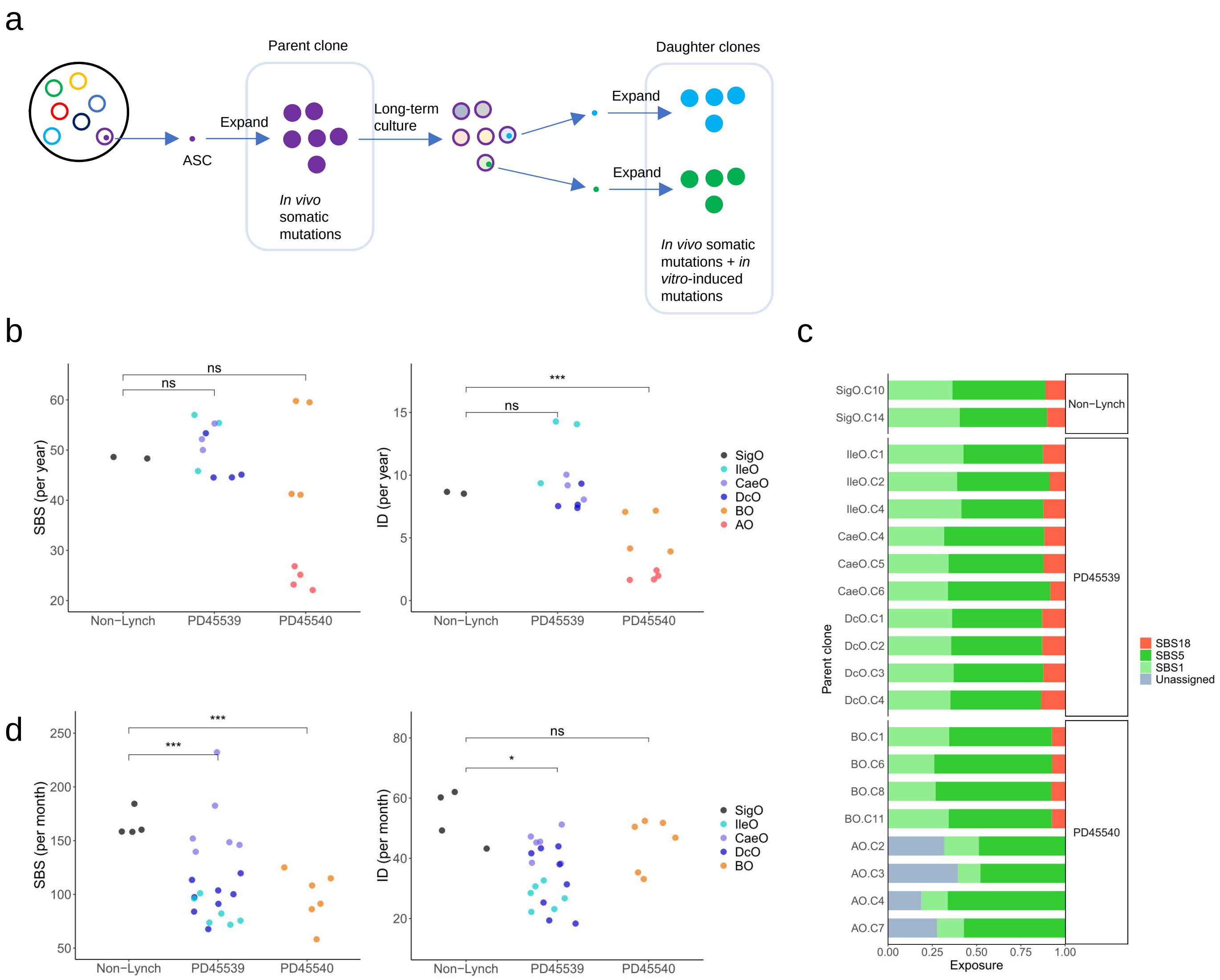

Supplementary Figure 3. Mutation burden of clonal organoids established from LS patients. a, Schematic diagram of clonal organoid establishment from LS patients. The early passage of organoids consisted of a pool of heterogenous clones. A clonal organoid grown from a single adult stem cell (ASC) was hand-picked and expanded into a parent clone. The genetically homogenous parent clone contained somatic mutations the ASC had accumulated *in vivo*. The parent clone was subjected to long-term culture, during which the ASCs accumulated mutations independent of each other. A second clonal step was performed at the end of the culturing period. Daughter clones were established from subcloned organoids. The daughter clones contained both *in vivo* somatic mutations and *in vitro*-induced mutations. **b,** Single base substitutions (SBS) and insertions and deletions (ID) burden in the parent clones. Each data point represents a parent clone and is colour-coded according to its tissue origin. Two-sided Wilcoxon test (SBS) and two-sided t-test (ID): ns = non-significant, PD45540 (p = 0.00052). SigO: Sigmoid organoid, IleO: Ileum organoid, CaeO: Caecum organoid, DcO: Descending colon organoid, BO: Body organoid, AO: Antrum organoid. **c,** Relative proportion of exposure of SBS mutational signatures in the parent clones. Unassigned signature refers to SBSs not explained by the HDP model and treated as noise. **d,** SBS and ID burden in the daughter clones. Each data point represents a daughter clone and is colour-coded according to its tissue origin. Two-sided t-test: ns = non-significant, PD45539 (p = 0.00028), PD45540 (p = 0.00044) for SBS; PD45539 (p = 0.014) for ID.

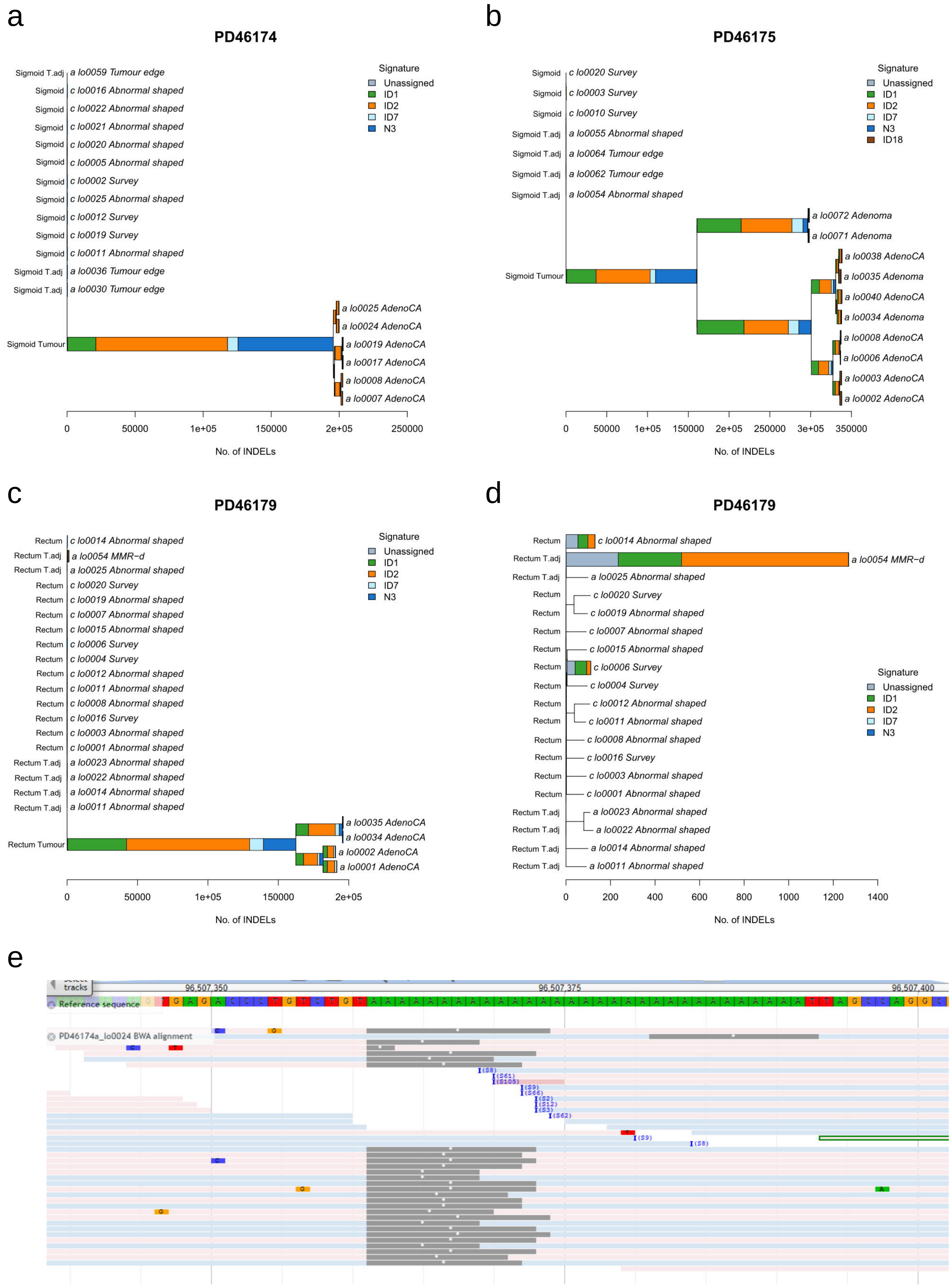

Supplementary Figure 4. ID mutational signatures in the tumour crypts of LS patients. a-c, Phylogenetic trees of PD46174, PD46175 and PD46179 mapped with ID mutational signatures. Branch lengths correspond to the number of INDELs. Each crypt is annotated with its identifier, crypt type and tissue origin. **d,** Phylogeny of non-neoplastic crypts in PD46179 after pruning off tumour crypts in **c**. **e,** Representative JBrowse screenshot of a single mutation which has likely arisen through the process responsible for indel signature N3.

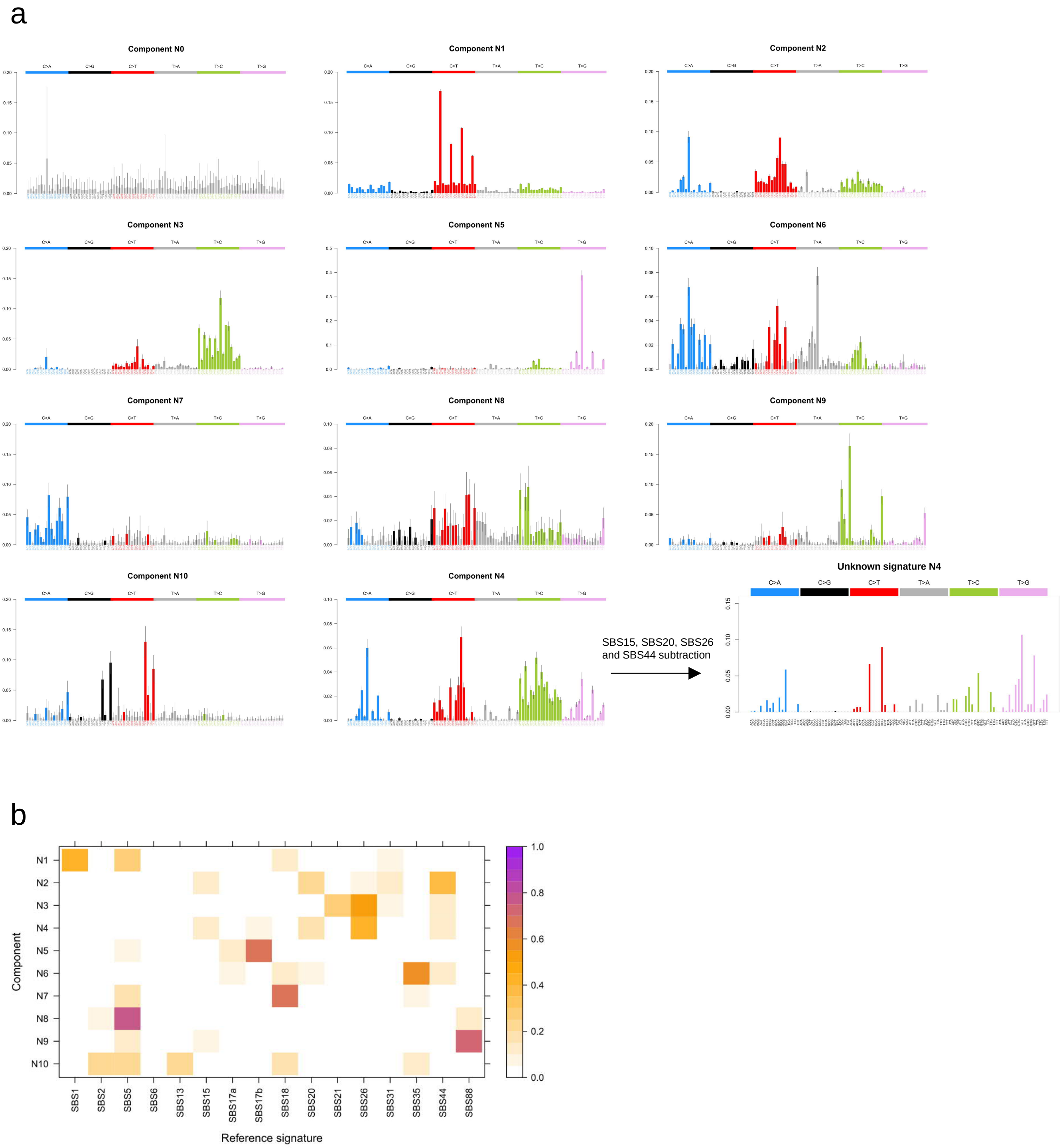
a
Component N0
Component N1
Component N2
Component N3
Component N5
Component N6
Component N7
Component N8
Component N9
Component N10
Component N4
Unknown signature N4
C>A
C>G
C>T
T>A
T>C
T>G
SBS15, SBS20, SBS26
and SBS44 subtraction
b
N1
N2
N3
N4
N5
N6
N7
N8
N9
N10
Component
SBS1
SBS2
SBS5
SBS6
SBS13
SBS15
SBS17a
SBS17b
SBS18
SBS20
SBS21
SBS26
SBS31
SBS35
SBS44
SBS88
Reference signature
1.0
0.8
0.6
0.4
0.2
0.0

c

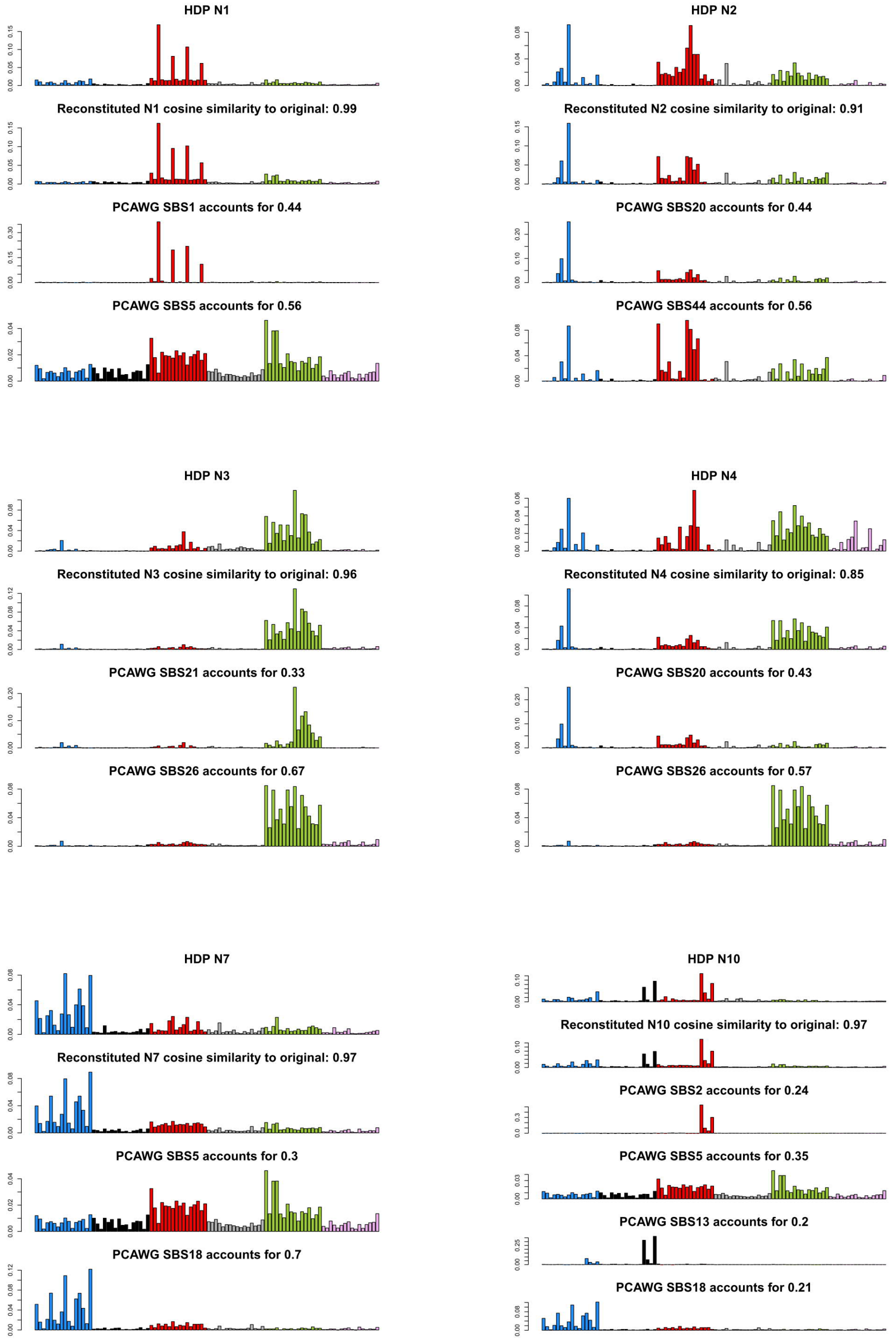

Supplementary Figure 5. SBS mutational signature extraction and deconvolution. a, 10 SBS components were extracted by HDP. Bar plots represent the mean values of each data category (96 trinucleotide combinations) after 2,500 iterations. Y-axis represents the relative contribution of SBSs. Error bars represent 95% credibility intervals. Non-significant data categories are shaded in light grey. Component N0 represented data unexplained by the model and was treated as noise. An unknown signature N4 was obtained after subtracting SBS15, SBS20, SBS26 and SBS44 from component N4. **b,** Heatmap showing the relative contribution of reference signatures to the 10 extracted SBS components. Component N5, N6, N8 and N9 corresponded to SBS17b, SBS35, SBS5 and SBS88, respectively. **c,** Mutation spectrums of component N1, N2, N3, N4, N7 and N10, and their reconstituted spectrums from deconvoluted signatures. Mutation spectrums are coloured according to the 6 classes of SBSs (from left to right: C>A, C>G, C>T, T>A, T>C and T>G). Y-axis represents the relative contribution of SBSs. N1 corresponded to a mixture of SBS1 and SBS5. N2 corresponded to a mixture of SBS20 and SBS44. N3 corresponded to a mixture of SBS21 and SBS26. N7 corresponded to a mixture of SBS5 and SBS18. N10 corresponded to a mixture of SBS2, SBS5, SBS13 and SBS18.

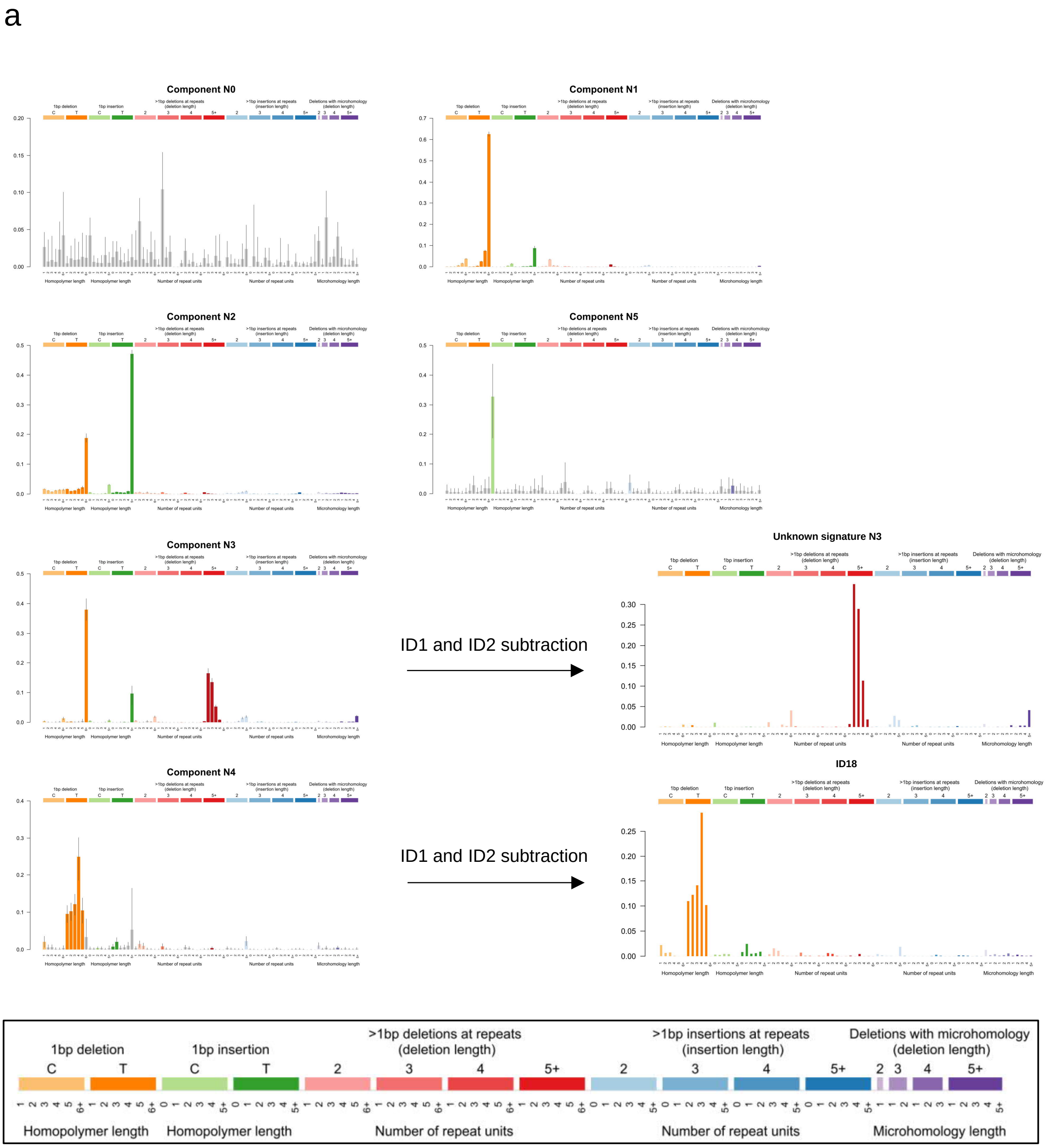
a
Component N0
Component N1
Component N2
Component N5
Component N3
Unknown signature N3
Component N4
ID18
ID1 and ID2 subtraction
ID1 and ID2 subtraction
1bp deletion
1bp insertion
>1bp deletions at repeats (deletion length)
>1bp insertions at repeats (insertion length)
Deletions with microhomology (deletion length)
C
T
C
T
2
3
4
5+
2
3
4
5+
2
3
4
5+
Homopolymer length
Homopolymer length
Number of repeat units
Number of repeat units
Microhomology length

b

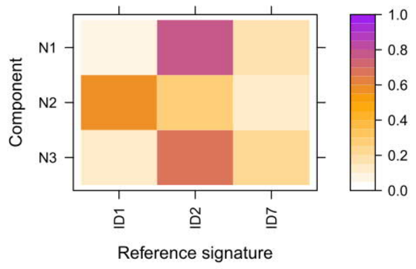

c

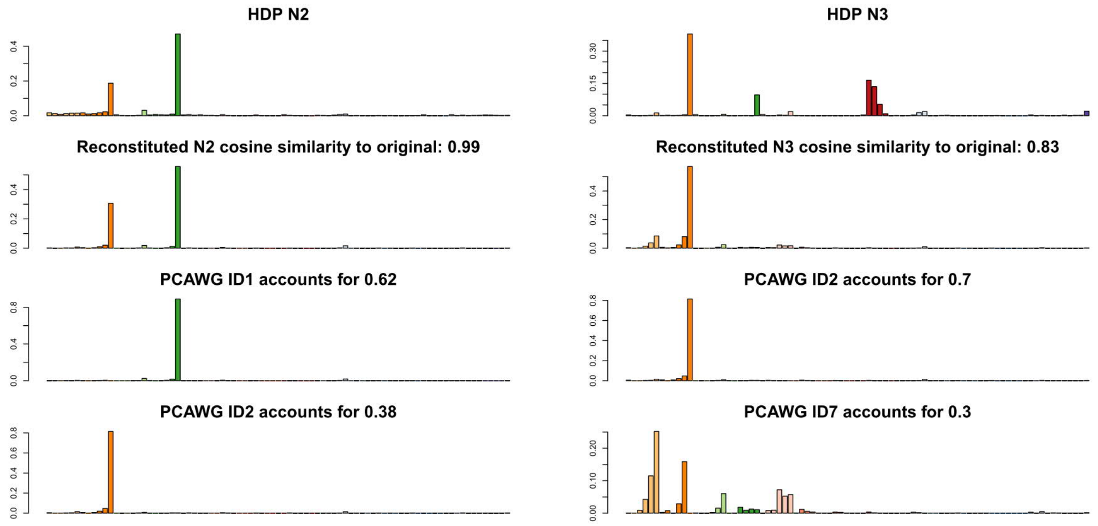

Supplementary Figure 6. ID mutational signature extraction and deconvolution. a, 5 ID components were extracted by HDP. INDEL data are coloured according to the length of insertions or deletions. Data with the same colour are sub-divided according to the number of repeating units in the underlying genome. Labels are enlarged in the legend at the bottom. Bar plots represent the mean values of each data category (83 INDEL combinations) after 2,500 iterations. Y-axis represents the relative contribution of INDELs. Error bars represent 95% credibility intervals. Non-significant data categories are shaded in light grey. Component N0 represented data unexplained by the model and was treated as noise. An unknown signature N3 was obtained after subtracting ID1 and ID2 from component N3. ID18 was obtained after subtracting ID1 and ID2 from component N4. Component N5 was absent in the 3 patients with tumours and was omitted from subsequent signature reassignment. **b,** Heatmap showing the relative contribution of reference signatures to ID component N1, N2 and N3. Component N1 corresponded to ID2. **c,** Mutation spectrums of component N2 and N3, and their reconstituted spectrums from deconvoluted signatures. Data are coloured according to the legend in a. N2 corresponded to a mixture of ID1 and ID2.

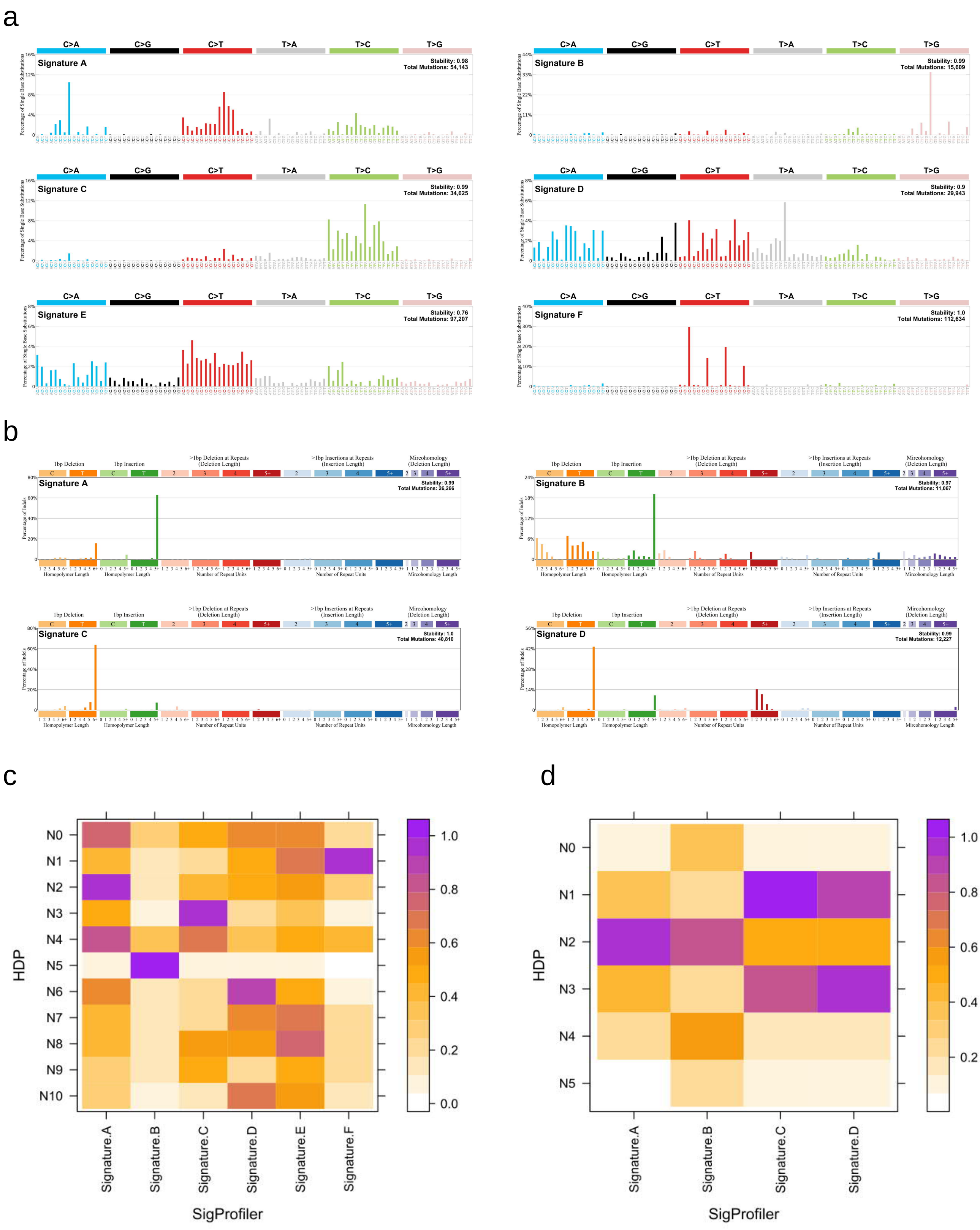

Supplementary Figure 7. Mutational signature validation. a, *De novo* SBS signatures extracted by SigProfiler. Signatures were plotted according to the relative proportion of the 96 trinucleotide contexts in the genome. **b,** *De novo* ID signatures extracted by SigProfiler. Signatures were plotted according to the relative proportion of the 83 insertion and deletion contexts in the genome. **c,** Heatmap showing the cosine similarity between SBS components extracted by HDP and SBS signatures extracted by SigProfiler. **d,** Heatmap showing the cosine similarity between ID components extracted by HDP and ID signatures extracted by SigProfiler.
